# Association of blood glucose change with postoperative delirium after coronary artery bypass grafting in patients with diabetes mellitus: a study of the MIMIC-IV database

**DOI:** 10.3389/fendo.2024.1400207

**Published:** 2024-06-20

**Authors:** Fujun Wang, Xue Mei

**Affiliations:** Department of Emergency, Beijing Chao-Yang Hospital, Capital Medical University, Beijing, China

**Keywords:** blood glucose, DM, CABG, LGMM model, POD

## Abstract

**Aim:**

Study results on blood glucose and the risk of delirium in patients receiving cardiac surgery are inconsistent, and there is also a gap in how to manage blood glucose after coronary artery bypass grafting (CABG). This study focused on patients with diabetes mellitus (DM) undergoing CABG and explored the associations of different blood glucose-related indexes and blood glucose change trajectory with postoperative delirium (POD), with the aim of providing some information for the management of blood glucose in this population.

**Methods:**

Data of patients with DM undergoing CABG were extracted from the Medical Information Mart for Intensive Care (MIMIC)-IV database in this retrospective cohort study. The blood glucose-related indexes included baseline blood glucose, mean blood glucose (MBG), mean absolute glucose (MAG), mean amplitude of glycemic excursions (MAGE), glycemic lability index (GLI), and largest amplitude of glycemic excursions (LAGE). The MBG trajectory was classified using the latent growth mixture modeling (LGMM) method. Univariate and multivariate logistic regression analyses were utilized to screen covariates and explore the associations of blood glucose-related indexes and MBG trajectory with POD. These relationships were also assessed in subgroups of age, gender, race, estimated glomerular filtration rate (eGFR), international normalized ratio (INR), sepsis, mechanical ventilation use, and vasopressor use. In addition, the potential interaction effect between blood glucose and hepatorenal function on POD was investigated. The evaluation indexes were odds ratios (ORs), relative excess risk due to interaction (RERI), attributable proportion of interaction (AP), and 95% confidence intervals (CIs).

**Results:**

Among the eligible 1,951 patients, 180 had POD. After adjusting for covariates, higher levels of MBG (OR = 3.703, 95% CI: 1.743–7.870), MAG >0.77 mmol/L/h (OR = 1.754, 95% CI: 1.235–2.490), and GLI >2.6 (mmol/L)^2^/h/per se (OR = 1.458, 95% CI: 1.033–2.058) were associated with higher odds of POD. The positive associations of MBG, MAG, and GLI with POD were observed in patients aged <65 years old, male patients, White patients, those with eGFR <60 and INR <1.5, patients with sepsis, and those who received mechanical ventilation and vasopressors (all *p* < 0.05). Patients with class 3 (OR = 3.465, 95% CI: 1.122–10.696) and class 4 (OR = 3.864, 95% CI: 2.083–7.170) MBG trajectory seemed to have higher odds of POD, compared to those with a class 1 MBG trajectory. Moreover, MAG (RERI = 0.71, 95% CI: 0.14–1.27, AP = 0.71, 95% CI: 0.12–1.19) and GLI (RERI = 0.78, 95% CI: 0.19–1.39, AP = 0.69, 95% CI: 0.16–1.12) both had a potential synergistic effect with INR on POD.

**Conclusion:**

Focusing on levels of MBG, MAG, GLI, and MBG trajectory may be more beneficial to assess the potential risk of POD than the blood glucose level upon ICU admission in patients with DM undergoing CABG.

## Introduction

Postoperative delirium (POD) is a serious complication of surgery characterized by an acute and fluctuating disorder of attention and cognition, which is associated with prolonged hospitalization, long-term cognitive decline, and mortality (1, 2). Coronary artery bypass grafting (CABG) is a common surgical treatment for cardiovascular diseases (CVDs) (3), and the risk of postoperative complications cannot be ignored, especially psychiatric symptoms (4, 5). Multiple studies have shown that patients receiving CAGB are more likely to develop delirium, with an incidence range between 8% and 54% (6, 7). In addition, diabetes mellitus (DM) is an established risk factor associated with significant morbidity and mortality after CABG and could be identified as an independent predictor of POD (8).

The pathophysiology of delirium is complex and heterogeneous. Disturbance of blood glucose metabolism has been identified as a risk factor for the onset of delirium (9). However, the association between blood glucose and delirium is yet to be determined to date. Zhao et al. (10) suggested that relative hypoglycemia is associated with mortality and the occurrence of delirium in critically ill patients with DM, whereas van Keulen et al. (9) believed that delirium was not linked to more pronounced glucose variability. A recent article showed that among patients with DM who underwent non-cardiac surgery, preoperative acute hyperglycemia was linked to POD, whereas chronic hyperglycemia had no significant association (11). Current studies on blood glucose and the risk of delirium in people receiving cardiac surgery are inconsistent, and there is also a gap in how to manage blood glucose after CABG. Intraoperative hyperinsulinemia–normoglycemia has been reported to augment the risk of delirium after cardiac surgery (12). In addition, intraoperative blood glucose fluctuation, manifested by intraoperative glycemic variability (GV), is associated with POD after cardiac surgery, and patients with a higher intraoperative GV have an increased risk of POD (13). In fact, existing studies have yet to explore the dynamic trajectory of blood glucose changes.

This study aims to explore the association of blood glucose with POD in patients with DM undergoing CABG on the basis of mean blood glucose (MBG), mean absolute glucose (MAG), mean amplitude of glycemic excursions (MAGE), glycemic lability index (GLI), largest amplitude of glycemic excursions (LAGE), and blood glucose trajectory in 24 h after intensive care unit (ICU) admission. We hope that the study results could provide some evidence on blood glucose management and POD risk reduction in this population in clinical practice.

## Methods

### Study design and participants

Data of participants were extracted from the Medical Information Mart for Intensive Care (MIMIC)-IV database in this retrospective cohort study. This database was jointly published by the Computational Physiology Laboratory of the Massachusetts Institute of Technology (MIT), Philips Medical, and Beth Israel Deaconess Medical Center (BIDMC) (14). Information on true hospital stays for patients admitted to a tertiary academic medical center in Boston, MA, USA was included in the MIMIC and intended to support a wide variety of research in healthcare. More details are available elsewhere: https://mimic.mit.edu/docs/.

There were 2,263 patients with DM who underwent CABG in the database. The exclusion criteria were as follows: (1) age <18 years old; (2) hospitalized in the ICU for less than 24 h; (3) comatose or diagnosed as delirium within 24 h after ICU admission; (4) diagnosed with dementia, psychoses, traumatic brain injury (TBI), reading disorder, intellectual disabilities, or neurological disease; and (5) with alcohol/drug abuse. Finally, 1,951 patients were eligible. The MIMIC database has been approved by the Institutional Review Boards (IRBs) of the MIT and the BIDMC. Since this database is publicly available, ethical approval has been waived by our hospital’s IRB.

### Definitions of DM and CABG

In the MIMIC-IV database, diagnosis of DM was according to the International Classification of Diseases, 9th revision (ICD-9) codes (begin with 250) or 10th revision (ICD-10) codes (beginning with E10–E14). Similarly, the ICD-9 codes (3611–3619 and 362) or ICD-10 codes (beginning with 0210–0213) were used to estimate the condition of CABG use. In addition, we only extracted the information of multiple-admission patients’ first records.

### Calculation of blood glucose-related indexes

To assess the blood glucose condition of these patients in 24 h after ICU admission, we calculated the MBG, MAG, MAGE, GLI, and LAGE based on their biochemical glucose records that were recorded every 4 h, namely, six records (T1–T6). To be specific, MBG was stratified as follows: no hyperglycemia (<140 mg/dL), mild hyperglycemia (140–200 mg/dL), and severe hyperglycemia (≥200 mg/dL), according to a previous study (15). MAG was calculated according to the following formula: MAG = ∑ (Δglucose)/∑ Δtime, and is expressed as mmol/L/h and mg/dL/h where appropriate (16). MAGE as the most typical GV index was defined as the average of the absolute values of all adjacent peak–valley differences greater than one standard deviation (17). MAG was divided into ≤0.77 mmol/L/h and >0.77 mmol/L/h, and MAGE was divided into ≤2.58 mmol/L and >2.58 mmol/L according to their median value, respectively. The GLI as the squared difference between consecutive glucose measurements per unit of actual time (Δh) between those records was calculated as follows: GLI = Σ [{Δglucose (mmol/L)}^2^·Δh −1] (per se) (18). GLI was stratified into ≤2.6 (mmol/L)^2^/h/per se and >2.6 (mmol/L)^2^/h/per se according to the median value. The LAGE stands for the glucose excursion for a certain duration, calculated using the formula LAGE = Glu_max_ − Glu_min_. In addition, LAGE was divided into two levels, namely, ≤79.2 mg/dL and >79.2 mg/dL (19).

### Construction of longitudinal trajectory of blood glucose

The latent growth mixture modeling (LGMM) was utilized to classify the 24-h trajectory of blood glucose in patients with DM undergoing CABG (20). To select the best number of potential categories, we built quadratic growth models containing one to five classes. Briefly, LGMM was fitted based on four conditions. First, the selected classes must have the smallest values of Akaike information criterion (AIC) and Bayesian information criterion (BIC), and have the largest log likelihood ratio. Second, the entropy should be >0.7. Third, the minimum sample size of each class should not be less than 1% of the total. Fourth, the average posterior probability of each class must be >70%.

### Variable selection

We also extracted variables as potential covariates from the MIMIC database, including age, gender, race, insurance, heart rate (HR), diastolic blood pressure (DBP), systolic blood pressure (SBP), temperature, SpO_2_, pH, white blood cell (WBC), red cell distribution width (RDW), platelet, hematocrit, estimate glomerular filtration rate (eGFR), international normalized ratio (INR), prothrombin time (PT), blood urea nitrogen (BUN), bicarbonate, sodium (Na), potassium (K), chloride, the Sequential Organ Failure Assessment (SOFA) score, the Charlson Comorbidity Index (CCI), the Glasgow Coma Scale (GCS), sepsis, CVD, chronic kidney disease (CKD), liver disease, depression, mechanical ventilation use, vasopressor use, sedative drug use, and antibiotic drug use.

### Outcome and follow-up duration

The study outcome was POD. Two steps were conducted to diagnose delirium. Firstly, if a patient with a Richmond Agitation and Sedation Scale (RASS) score <−3 was recognized as coma, the patient was not suitable for subsequent assessment (21). Secondly, among eligible patients (with RASS score ≥−3), delirium was evaluated through the Confusion Assessment Method for the ICU (CAM-ICU) (22). Specifically, the CAM-ICU consists of four features: Feature 1: altered level of consciousness (LOC) (MIMIC-IV item ID 228334), Feature 2: multiple sclerosis (MS) change (ID 228337, 228300, and 229326), Feature 3: inattention (ID 228301 and 228336), and Feature 4: disorganized thinking (ID 228335, 228303, and 229324). Individuals who gave positive answers to Features 1 and 2, and to Feature 3 or Feature 4 were diagnosed with delirium.

The MIMIC was followed up by information in the electronic medical charts and hospital department records or by making contact with the patients, their family members, their attending healthcare workers, or family physicians on the phone. The follow-up started from 24 h after ICU admission to the occurrence of delirium or upon discharge.

### Statistical analysis

Continuous data were described using median and quartiles [M (Q_1_, Q_3_)], and the Mann–Whitney test was utilized for the comparison between the POD group and the non-POD group. Frequency and composition ratio [*N* (%)] was employed to describe the distribution of categorical data, and the chi-square test (χ²) was used for comparison. Box plots were drawn to reflect the blood glucose condition between the non-POD group and the POD group, and that among four selected classes in LGMM, using the Wilcoxon rank sum test. The LGMM was constructed using the R package “LCMM” (https://www.jstatsoft.org/article/view/v078i02). Univariate logistic regression analysis was used to screen covariates. Univariate and multivariate logistic regression analyses were utilized to investigate the association of blood glucose condition in 24 h after ICU admission (including baseline blood glucose, MBG, MAG, MAGE, GLI, LAGE, and MBG trajectories) with POD in patients with DM undergoing CABG. Model 1 was the unadjusted model. Model 2 was adjusted for age, HR, and SpO_2_. Model 3 was adjusted for age, HR, SpO_2_, RDW, hematocrit, eGFR, INR, PT, BUN, bicarbonate, and Na. Model 4 was adjusted for age, HR, SpO_2_, RDW, hematocrit, eGFR, INR, PT, BUN, bicarbonate, Na, SOFA, and CCI. Model 5 was adjusted for age, HR, SpO_2_, RDW, hematocrit, eGFR, INR, PT, BUN, bicarbonate, Na, SOFA, CCI, sepsis, CKD, mechanical ventilation status, and vasopressor use. These associations were also explored in subgroups of age, gender, race, eGFR, INR, sepsis, mechanical ventilation use, and vasopressor use. The evaluation indexes were odds ratios (ORs) and 95% confidence intervals (CIs). *p* < 0.05 indicates significant difference. In addition, potential interaction effects between blood glucose condition and INR and eGFR were assessed. Relative excess risk due to interaction (RERI) and attributable proportion of interaction (AP) were used to evaluate these interaction effects. When the CIs of RERI and AP contained 0, there was no significant interaction effect. Statistical analyses were conducted by R version 4.2.1. (2022–06-23 ucrt).

## Results

### Characteristics of patients with DM undergoing CABG

**Figure 1:**
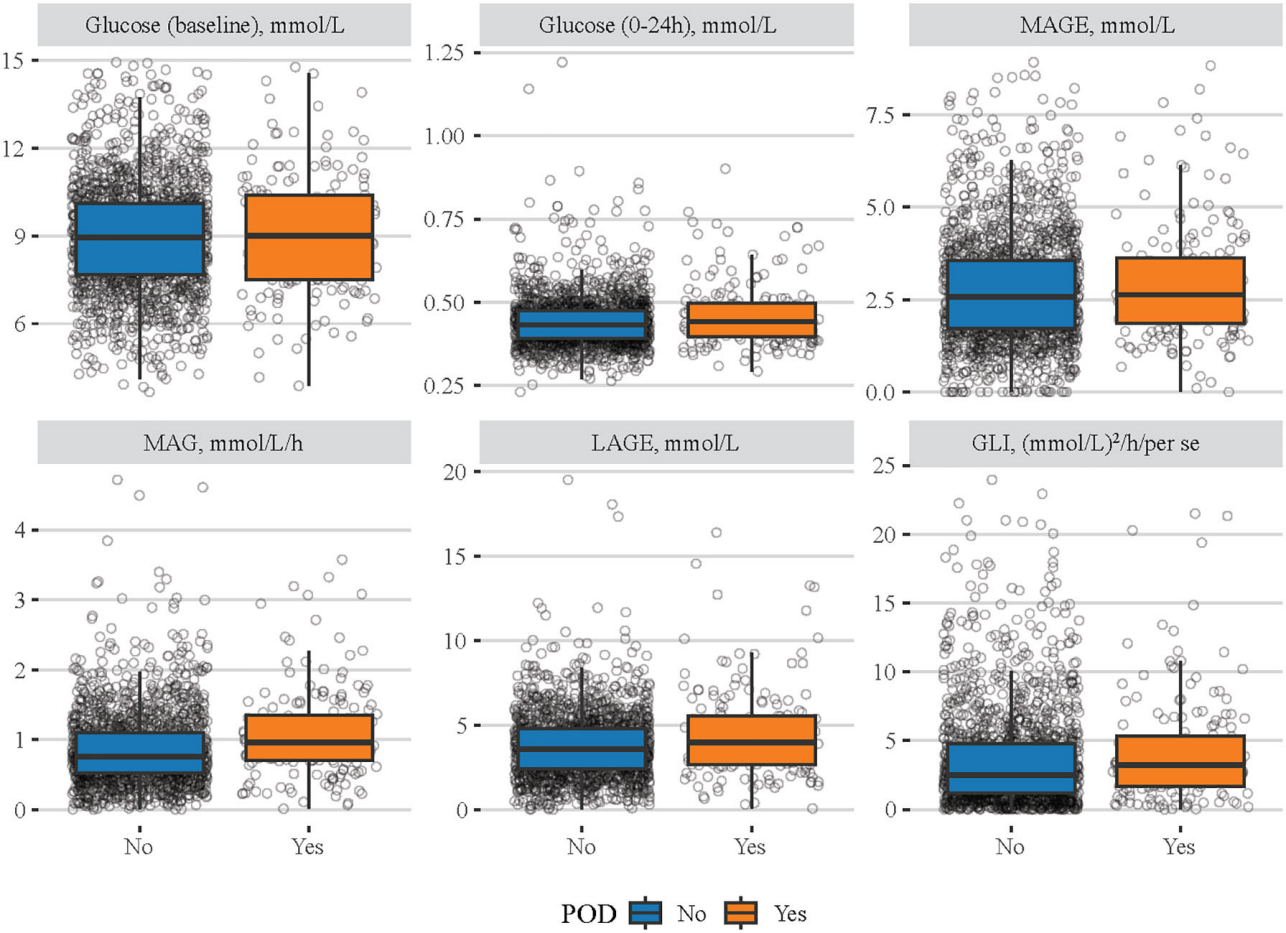
Difference of different blood glucose-related indexes between the non-POD group and the POD group. The blue boxes represent the non-POD group and the orange boxes denote the POD group.

The characteristics of eligible patients between the non-POD group and the POD group are shown in **Table 1**. Among eligible patients, 180 had POD. The median age of the total population was 68 years old, and 1,443 (73.96%) were men. Although the median baseline blood pressure was not significantly different between the non-POD group and the POD group, the median of MBG (140.00 mg/dL vs. 143.28 mg/dL), LAGE (64.60 mg/dL vs. 72.50 mg/dL), MAG (0.76 mmol/L/h vs. 0.97 mmol/L/h), and GLI [2.54 (mmol/L)^2^/h/per se vs. 3.27 (mmol/L)^2^/h/per se] was significantly lower in non-POD patients than in POD patients. Similarly, **Figure 1** clearly shows the difference of these indexes between the non-POD group and POD group, and details on statistical values are shown in **Supplementary Table S1**. In addition, age, insurance, HR, SpO_2_, RDW, hematocrit, eGFR, INR, PT, BUN, bicarbonate, Na, SOFA, CCI, sepsis, CKD, mechanical ventilation use, vasopressor use, and antibiotic drug use were significantly different between these two groups (all *p* < 0.05).

**Table 1:** Characteristics of patients with DM who underwent CABG between the non-POD group and the POD group.

Variables	Total (*n* = 1,951)	Non-POD (*n* = 1,771)	POD (*n* = 180)	Statistic	*p*
Age, years, M (Q_1_, Q_3_)	68.00 (61.00, 74.00)	67.00 (60.00, 73.00)	71.00 (64.00, 77.00)	*Z* = −4.995	**<0.001**
Gender, *n* (%)				χ² = 3.262	0.071
Female	508 (26.04)	451 (25.47)	57 (31.67)		
Male	1,443 (73.96)	1,320 (74.53)	123 (68.33)		
Race, *n* (%)				χ² = 0.456	0.499
White	1,344 (68.89)	1,224 (69.11)	120 (66.67)		
Other	607 (31.11)	547 (30.89)	60 (33.33)		
Insurance, *n* (%)				χ² = 7.976	**0.019**
Medicaid	81 (4.15)	73 (4.12)	8 (4.44)		
Medicare	885 (45.36)	786 (44.38)	99 (55.00)		
Other	985 (50.49)	912 (51.50)	73 (40.56)		
HR, bpm, M (Q_1_, Q_3_)	80.00 (76.00, 87.00)	80.00 (75.00, 87.00)	83.00 (80.00, 91.00)	*Z* = −5.358	**<0.001**
DBP, mmHg, M (Q_1_, Q_3_)	57.00 (51.00, 64.00)	57.00 (51.50, 65.00)	56.50 (49.00, 63.00)	*Z* = −1.741	0.082
SBP, mmHg, M (Q_1_, Q_3_)	112.00 (102.00, 125.00)	113.00 (102.00, 125.00)	110.00 (99.00, 123.25)	*Z* = −1.399	0.162
Temperature, M (Q_1_, Q_3_)	36.44 (36.06, 36.70)	36.44 (36.10, 36.70)	36.44 (35.90, 36.72)	*Z* = −0.229	0.819
SpO_2_, %, M (Q_1_, Q_3_)	100.00 (99.00, 100.00)	100.00 (99.00, 100.00)	100.00 (98.00, 100.00)	*Z* = −3.092	**0.002**
pH, M (Q_1_, Q_3_)	7.40 (7.37, 7.44)	7.40 (7.37, 7.44)	7.40 (7.35, 7.44)	*Z* = −0.839	0.401
WBC, K/UL, M (Q_1_, Q_3_)	12.40 (9.30, 15.80)	12.40 (9.20, 15.70)	12.80 (9.65, 16.38)	*Z* = −1.089	0.276
RDW, %, M (Q_1_, Q_3_)	13.60 (13.00, 14.50)	13.60 (13.00, 14.40)	14.00 (13.30, 15.10)	*Z* = −4.766	**<0.001**
Platelet, K/UL, M (Q_1_, Q_3_)	151.00 (122.00, 191.00)	151.00 (124.00, 190.00)	145.50 (110.75, 192.50)	*Z* = −1.728	0.084
Hematocrit, %, M (Q_1_, Q_3_)	29.00 (24.00, 34.00)	29.00 (25.00, 34.00)	26.00 (23.00, 31.00)	*Z* = −4.652	**<0.001**
eGFR, mL/min/1.73 m^2^, M (Q_1_, Q_3_)	83.28 (64.88, 96.19)	84.74 (66.54, 96.84)	72.69 (47.55, 86.06)	*Z* = −6.178	**<0.001**
INR, M (Q_1_, Q_3_)	1.40 (1.20, 1.50)	1.40 (1.20, 1.50)	1.50 (1.30, 1.60)	*Z* = −4.626	**<0.001**
PT, s, M (Q_1_, Q_3_)	15.00 (13.80, 16.50)	15.00 (13.80, 16.40)	15.90 (14.00, 17.80)	*Z* = −4.175	**<0.001**
BUN, mg/dL, M (Q_1_, Q_3_)	17.00 (13.00, 23.00)	17.00 (13.00, 22.00)	20.50 (15.00, 27.25)	*Z* = −4.481	**<0.001**
Bicarbonate, mEq/L, M (Q_1_, Q_3_)	23.00 (22.00, 25.00)	23.00 (22.00, 25.00)	22.00 (21.00, 24.00)	*Z* = −5.214	**<0.001**
Na, mEq/L, M (Q_1_, Q_3_)	136.00 (134.00, 137.00)	136.00 (134.00, 137.00)	135.00 (133.00, 137.00)	*Z* = −2.153	**0.031**
K, mEq/L, M (Q_1_, Q_3_)	4.60 (4.10, 5.10)	4.60 (4.10, 5.10)	4.70 (4.20, 5.23)	*Z* = −1.387	0.165
Chloride, mEq/L, M (Q_1_, Q_3_)	106.00 (104.00, 108.00)	106.00 (104.00, 108.00)	106.00 (103.00, 109.00)	*Z* = −0.244	0.807
SOFA, M (Q_1_, Q_3_)	3.00 (1.00, 4.00)	3.00 (1.00, 4.00)	4.00 (2.00, 6.00)	*Z* = −5.664	**<0.001**
CCI, M (Q_1_, Q_3_)	3.00 (2.00, 4.00)	3.00 (2.00, 4.00)	4.00 (3.00, 6.00)	*Z* = −8.528	**<0.001**
GCS, M (Q_1_, Q_3_)	15.00 (15.00, 15.00)	15.00 (15.00, 15.00)	15.00 (15.00, 15.00)	*Z* = −0.351	0.726
Sepsis, *n* (%)				χ² = 7.403	**0.007**
No	1,171 (60.02)	1,080 (60.98)	91 (50.56)		
Yes	780 (39.98)	691 (39.02)	89 (49.44)		
CVD, *n* (%)				χ² = 3.182	0.074
No	282 (14.45)	264 (14.91)	18 (10.00)		
Yes	1,669 (85.55)	1,507 (85.09)	162 (90.00)		
CKD, *n* (%)				χ² = 20.353	**<0.001**
No	1,535 (78.68)	1,417 (80.01)	118 (65.56)		
Yes	416 (21.32)	354 (19.99)	62 (34.44)		
Liver disease, *n* (%)				χ² = 1.143	0.285
No	1,881 (96.41)	1,710 (96.56)	171 (95.00)		
Yes	70 (3.59)	61 (3.44)	9 (5.00)		
Depression, *n* (%)				χ² = 1.437	0.231
No	1,849 (94.77)	1,675 (94.58)	174 (96.67)		
Yes	102 (5.23)	96 (5.42)	6 (3.33)		
Mechanical ventilation use, *n* (%)				χ² = 27.680	**<0.001**
No	768 (39.36)	730 (41.22)	38 (21.11)		
Yes	1,183 (60.64)	1,041 (58.78)	142 (78.89)		
Vasopressor use, *n* (%)				χ² = 22.685	**<0.001**
No	366 (18.76)	356 (20.10)	10 (5.56)		
Yes	1,585 (81.24)	1,415 (79.90)	170 (94.44)		
Sedative drug use, *n* (%)				χ² = 0.447	0.504
No	53 (2.72)	50 (2.82)	3 (1.67)		
Yes	1,898 (97.28)	1,721 (97.18)	177 (98.33)		
Antibiotic drug use, *n* (%)				χ² = 4.017	**0.045**
No	58 (2.97)	57 (3.22)	1 (0.56)		
Yes	1,893 (97.03)	1,714 (96.78)	179 (99.44)		
Baseline blood glucose, mg/dL, M (Q_1_, Q_3_)	162.00 (138.50, 184.25)	161.67 (138.68, 183.42)	165.62 (135.47, 192.75)	*Z* = −1.414	0.157
MBG, mg/dL, M (Q_1_, Q_3_)	140.25 (127.40, 154.75)	140.00 (127.31, 153.90)	143.28 (128.62, 161.13)	*Z* = −2.230	**0.026**
LAGE, mg/dL, M (Q_1_, Q_3_)	65.00 (44.25, 88.00)	64.60 (43.75, 87.29)	72.50 (48.88, 100.10)	*Z* = −3.466	**<0.001**
MAG, mmol/L/h, M (Q_1_, Q_3_)	0.78 (0.53, 1.13)	0.76 (0.52, 1.10)	0.97 (0.70, 1.37)	*Z* = −5.209	**<0.001**
MAGE, mmol/L, M (Q_1_, Q_3_)	2.58 (1.75, 3.60)	2.57 (1.74, 3.59)	2.64 (1.92, 3.76)	*Z* = −1.234	0.217
GLI, (mmol/L)^2^/h/per se, M (Q_1_, Q_3_)	2.60 (1.21, 4.97)	2.54 (1.19, 4.89)	3.27 (1.82, 6.35)	*Z* = −3.454	**<0.001**
Baseline blood glucose, *n* (%)				χ² = 7.930	**0.019**
No hyperglycemia	510 (26.14)	462 (26.09)	48 (26.67)		
Mild hyperglycemia	1,169 (59.92)	1,074 (60.64)	95 (52.78)		
Severe hyperglycemia	272 (13.94)	235 (13.27)	37 (20.56)		
MBG, *n* (%)				χ² = 23.478	**<0.001**
No hyperglycemia	964 (49.41)	885 (49.97)	79 (43.89)		
Mild hyperglycemia	922 (47.26)	838 (47.32)	84 (46.67)		
Severe hyperglycemia	65 (3.33)	48 (2.71)	17 (9.44)		
LAGE, mg/dL, *n* (%)				χ² = 11.165	**<0.001**
≤79.2	1,292 (66.22)	1,193 (67.36)	99 (55.00)		
>79.2	659 (33.78)	578 (32.64)	81 (45.00)		
GLI, (mmol/L)^2^/h/per se, *n* (%)				χ² = 17.786	**<0.001**
≤2.6	975 (49.97)	912 (51.50)	63 (35.00)		
>2.6	976 (50.03)	859 (48.50)	117 (65.00)		
MAG, mmol/L/h, *n* (%)				χ² = 28.223	**<0.001**
≤0.77	975 (49.97)	919 (51.89)	56 (31.11)		
>0.77	976 (50.03)	852 (48.11)	124 (68.89)		
MAGE, mmol/L, *n* (%)				χ² = 0.601	0.438
≤2.58	975 (49.97)	890 (50.25)	85 (47.22)		
>2.58	976 (50.03)	881 (49.75)	95 (52.78)		
LGMM classes, *n* (%)				-	**<0.001**
1	1,711 (87.7)	1,570 (88.65)	141 (78.33)		
2	126 (6.46)	116 (6.55)	10 (5.56)		
3	26 (1.33)	19 (1.07)	7 (3.89)		
4	88 (4.51)	66 (3.73)	22 (12.22)		

Z: Mann–Whitney test, χ²: chi-square test.

DM, diabetes mellitus; CABG, coronary artery bypass grafting; POD, postoperative delirium; M, median; Q_1_, 1st quartile; Q_3_, 3rd quartile; HR, heart rate; DBP, diastolic blood pressure; SBP, systolic blood pressure; WBC, white blood cell; RDW, red cell distribution width; eGFR, estimate glomerular filtration rate; INR, international normalized ratio; PT, prothrombin time; BUN, blood urea nitrogen; Na, sodium; K, potassium; SOFA, Sequential Organ Failure Assessment; CCI, Charlson Comorbidity Index; GCS, Glasgow Coma Scale; CVD, cardiovascular disease; CKD, chronic kidney disease; MBG, mean blood glucose; LAGE, largest amplitude of glycemic excursions; GLI, glycemic lability index; MAG, mean absolute glucose; MAGE, mean amplitude of glycemic excursions; LGMM, latent growth mixture modeling.

The bold values provided means statistically significant (namely P<0.05).

### Association of blood glucose conditions with POD

Before we investigated the associations of blood glucose-related indexes with POD in patients with DM undergoing CABG, the covariates were screened (**Supplementary Table S2**). The results showed that age, HR, SpO_2_, RDW, hematocrit, eGFR, INR, PT, BUN, bicarbonate, Na, SOFA, CCI, sepsis, CKD, mechanical ventilation use, and vasopressor use were significantly associated with POD (all *p* < 0.05).

Then, we explored the associations of different blood glucose indexes with POD (**Table 2**). After adjusting for all selected covariates, patients who had a higher MBG level (severe hyperglycemia) seemed to have higher odds of POD compared with those who had no hyperglycemia (OR = 3.703, 95% CI: 1.743–7.870). Patients with MAG levels >0.77 mmol/L/h (OR = 1.754, 95% CI: 1.235–2.490) or GLI >2.6 (mmol/L)^2^/h/per (OR = 1.458, 95% CI: 1.033–2.058) had higher odds of POD compared to MGA level ≤0.77 mmol/L/h or GLI ≤2.6 (mmol/L)^2^/h/per, respectively.

**Table 2:** Associations of different blood glucose indexes with POD in patients with DM who underwent CABG.

Variables	Model 1		Model 2		Model 3		Model 4		Model 5	
	OR (95% CI)	*p*	OR (95% CI)	*p*	OR (95% CI)	*p*	OR (95% CI)	*p*	OR (95% CI)	*p*
Baseline blood glucose										
No hyperglycemia	Ref		Ref		Ref		Ref		Ref	
Mild hyperglycemia	0.851 (0.592–1.225)	0.386	0.870 (0.601–1.261)	0.463	0.843 (0.571–1.244)	0.390	0.825 (0.557–1.221)	0.336	0.834 (0.562–1.237)	0.367
Severe hyperglycemia	1.515 (0.960–2.393)	0.074	1.262 (0.779–2.044)	0.344	1.205 (0.726–2.000)	0.470	1.188 (0.715–1.974)	0.507	1.206 (0.723–2.012)	0.472
MBG										
No hyperglycemia	Ref		Ref		Ref		Ref		Ref	
Mild hyperglycemia	1.123 (0.814–1.549)	0.480	1.111 (0.797–1.548)	0.535	1.192 (0.844–1.683)	0.320	1.138 (0.802–1.614)	0.468	1.181 (0.831–1.680)	0.353
Severe hyperglycemia	3.968 (2.179–7.223)	<0.001	2.958 (1.513–5.784)	0.002	3.470 (1.655–7.277)	<0.001	3.843 (1.825–8.094)	<0.001	3.703 (1.743–7.870)	**<0.001**
MAG										
≤0.77	Ref		Ref		Ref		Ref		Ref	
>0.77	2.388 (1.719–3.319)	<0.001	2.233 (1.595–3.126)	<0.001	1.840 (1.300–2.604)	<0.001	1.766 (1.245–2.504)	0.001	1.754 (1.235–2.490)	**0.002**
MAGE										
≤2.58	Ref		Ref		Ref		Ref		Ref	
>2.58	1.129 (0.831–1.535)	0.438	1.130 (0.825–1.548)	0.446	1.031 (0.744–1.428)	0.855	1.022 (0.736–1.419)	0.895	1.022 (0.735–1.420)	0.898
LAGE										
≤79.2	Ref		Ref		Ref		Ref		Ref	
>79.2	1.689 (1.239–2.302)	<0.001	1.572 (1.142–2.164)	0.005	1.376 (0.987–1.917)	0.060	1.326 (0.949–1.852)	0.098	1.328 (0.949–1.859)	0.098
GLI										
≤2.6	Ref		Ref		Ref		Ref		Ref	
>2.6	1.972 (1.432–2.716)	<0.001	1.873 (1.350–2.601)	<0.001	1.516 (1.078–2.134)	0.017	1.467 (1.041–2.068)	0.029	1.458 (1.033–2.058)	**0.032**

POD, postoperative delirium; DM, diabetes mellitus; CABG, coronary artery bypass grafting; OR, odds ratio; CI, confidence interval; Ref, reference; MBG, mean blood glucose; MAG, mean absolute glucose; MAGE, mean amplitude of glycemic excursions; LAGE, largest amplitude of glycemic excursions; GLI, glycemic lability index.

Model 1: unadjusted model.

Model 2: adjusted for age, HR, and SpO_2_.

Model 3: adjusted for age, HR, SpO_2_, RDW, hematocrit, eGFR, INR, PT, BUN, bicarbonate, and Na.

Model 4: adjusted for age, HR, SpO_2_, RDW, hematocrit, eGFR, INR, PT, BUN, bicarbonate, Na, SOFA, and CCI.

Model 5: adjusted for age, HR, SpO_2_, RDW, hematocrit, eGFR, INR, PT, BUN, bicarbonate, Na, SOFA, CCI, sepsis, CKD, mechanical ventilation, use and vasopressor use.

The bold values provided means statistically significant (namely P<0.05).

The associations of MBG, MAG, and GLI with POD were further assessed in subgroups of age, gender, race, eGFR, INR, sepsis, mechanical ventilation use, and vasopressor use (**Table 3**). After adjusting for covariates, higher levels of MBG, MAG, and GLI were associated with higher odds of POD in patients aged <65 years old, male patients, White patients, patients with eGFR <60 and INR <1.5, those with sepsis, and those who received mechanical ventilation or vasopressors (all *p* < 0.05). The positive association between MBG and POD was observed in patients aged ≥65 years old, female patients, patients from other races, those with eGFR ≥60, and those from the non-ventilation subgroup (all *p* < 0.05). Moreover, only patients aged ≥65 years old and those with eGFR ≥60 and MAG >0.77 mmol/L/h were associated with higher odds of POD (all *p* < 0.05).

**Table 3:** Associations of MBG, MAG, and GLI with POD in different subgroups.

Variables	OR (95% CI)	*p*	OR (95% CI)	*p*
	Age <65 years old		Age ≥65 years old	
MBG				
No hyperglycemia	Ref		Ref	
Mild hyperglycemia	1.188 (0.623–2.263)	0.601	1.187 (0.789–1.785)	0.410
Severe hyperglycemia	3.983 (1.454–10.912)	**0.007**	3.874 (1.481–10.133)	**0.006**
MAG				
≤0.77	Ref		Ref	
>0.77	3.139 (1.599–6.162)	**<0.001**	1.588 (1.060–2.378)	**0.025**
GLI				
≤2.6	Ref		Ref	
>2.6	2.225 (1.180–4.195)	**0.013**	1.389 (0.930–2.074)	0.108
	Female		Male	
MBG				
No hyperglycemia	Ref		Ref	
Mild hyperglycemia	1.584 (0.792–3.169)	0.193	1.016 (0.666–1.550)	0.942
Severe hyperglycemia	7.077 (1.659–30.195)	**0.008**	2.628 (1.061–6.509)	**0.037**
MAG				
≤0.77	Ref		Ref	
>0.77	1.274 (0.656–2.477)	0.474	2.055 (1.344–3.141)	**<0.001**
GLI				
≤2.6	Ref		Ref	
>2.6	1.054 (0.546–2.034)	0.876	1.716 (1.134–2.596)	**0.011**
	White		Others	
MBG				
No hyperglycemia	Ref		Ref	
Mild hyperglycemia	1.200 (0.783–1.840)	0.402	1.202 (0.632–2.286)	0.575
Severe hyperglycemia	3.135 (1.237–7.944)	**0.016**	5.672 (1.569–20.503)	**0.008**
MAG				
≤0.77	Ref		Ref	
>0.77	1.934 (1.256–2.977)	**0.003**	1.423 (0.765–2.646)	0.265
GLI				
≤2.6	Ref		Ref	
>2.6	1.660 (1.085–2.540)	**0.020**	1.160 (0.627–2.147)	0.635
	eGFR ≥ 60		eGFR < 60	
MBG				
No hyperglycemia	Ref		Ref	
Mild hyperglycemia	1.341 (0.873–2.060)	0.180	1.072 (0.561–2.047)	0.834
Severe hyperglycemia	4.627 (1.720–12.444)	**0.002**	3.515 (1.043–11.839)	**0.042**
MAG				
≤0.77	Ref		Ref	
>0.77	1.605 (1.055–2.441)	**0.027**	2.111 (1.098–4.057)	**0.025**
GLI				
≤2.6	Ref		Ref	
>2.6	1.345 (0.893–2.026)	0.157	2.025 (1.041–3.935)	**0.038**
	INR < 1.5		INR ≥ 1.5	
MBG				
No hyperglycemia	Ref		Ref	
Mild hyperglycemia	1.138 (0.688–1.883)	0.615	1.197 (0.727–1.971)	0.479
Severe hyperglycemia	4.035 (1.573–10.355)	**0.004**	1.381 (0.326–5.848)	0.661
MAG	Ref		Ref	
≤0.77	3.392 (1.980–5.810)	**<0.001**	0.935 (0.569–1.538)	0.792
>0.77				
GLI	Ref		Ref	
≤2.6	2.635 (1.573–4.413)	**<0.001**	0.787 (0.480–1.289)	0.341
	Non-sepsis		Sepsis	
MBG				
No hyperglycemia	Ref		Ref	
Mild hyperglycemia	1.065 (0.659–1.721)	0.797	1.248 (0.735–2.117)	0.412
Severe hyperglycemia	2.919 (0.810–10.524)	0.101	3.729 (1.348–10.322)	**0.011**
MAG				
≤0.77	Ref		Ref	
>0.77	1.481 (0.921–2.381)	0.105	2.038 (1.193–3.482)	**0.009**
GLI				
≤2.6	Ref		Ref	
>2.6	1.125 (0.703–1.799)	0.624	1.939 (1.150–3.271)	**0.013**
	Non-ventilation		Mechanical ventilation	
MBG				
No hyperglycemia	Ref		Ref	
Mild hyperglycemia	1.495 (0.681–3.279)	0.316	1.107 (0.772–1.587)	0.579
Severe hyperglycemia	7.338 (2.200–24.474)	**0.001**	5.384 (2.123–13.652)	**<0.001**
MAG				
≤0.77	Ref		Ref	
>0.77	1.548 (0.769–3.115)	0.221	2.443 (1.653–3.612)	**<0.001**
GLI				
≤2.6	Ref		Ref	
>2.6	1.300 (0.642–2.632)	0.465	1.965 (1.350–2.862)	**<0.001**
	Non-vasopressors		Vasopressors	
MBG				
No hyperglycemia	Ref		Ref	
Mild hyperglycemia	9.206 (1.033–82.069)	**0.047**	1.101 (0.790–1.535)	0.570
Severe hyperglycemia	10.282 (0.458–230.850)	0.142	6.141 (3.075–12.262)	**<0.001**
MAG				
≤0.77	Ref		Ref	
>0.77	2.885 (0.566–14.706)	0.202	2.222 (1.577–3.129)	**<0.001**
GLI				
≤2.6	Ref		Ref	
>2.6	2.900 (0.552–15.242)	0.209	1.879 (1.347–2.620)	**<0.001**

MBG, mean blood glucose; MAG, mean absolute glucose; GLI, glycemic lability index; POD, postoperative delirium; OR, odds ratio; CI, confidence interval; Ref, reference.

Age subgroups: adjusted for HR, SpO_2_, RDW, hematocrit, eGFR, INR, PT, BUN, bicarbonate, Na, SOFA, CCI, sepsis, CKD, mechanical ventilation use, and vasopressor use.

Gender subgroups: adjusted for age, HR, SpO_2_, RDW, hematocrit, eGFR, INR, PT, BUN, bicarbonate, Na, SOFA, CCI, sepsis, CKD, mechanical ventilation use, and vasopressor use.

Race subgroups: adjusted for age, HR, SpO_2_, RDW, hematocrit, eGFR, INR, PT, BUN, bicarbonate, Na, SOFA, CCI, sepsis, CKD, mechanical ventilation use, and vasopressor use.

eGFR subgroups: age, HR, SpO_2_, RDW, hematocrit, INR, PT, BUN, bicarbonate, Na, SOFA, CCI, sepsis, CKD, mechanical ventilation use, and vasopressor use.

INR subgroups: adjusted for age, HR, SpO_2_, RDW, hematocrit, eGFR, PT, BUN, bicarbonate, Na, SOFA, CCI, sepsis, CKD, mechanical ventilation use, and vasopressor use.

Sepsis subgroups: adjusted for age, HR, SpO_2_, RDW, hematocrit, eGFR, INR, PT, BUN, bicarbonate, Na, SOFA, CCI, CKD, mechanical ventilation use, and vasopressor use.

Mechanical ventilation use subgroups: adjusted for age, HR, SpO_2_, RDW, hematocrit, eGFR, INR, PT, BUN, bicarbonate, Na, SOFA, CCI, sepsis, CKD, and vasopressor use.

Vasopressor use subgroups: adjusted for age, HR, SpO_2_, RDW, hematocrit, eGFR, INR, PT, BUN, bicarbonate, Na, SOFA, CCI, sepsis, CKD, and mechanical ventilation use.

The bold values provided means statistically significant (namely P<0.05).

### Association between blood glucose trajectories and POD in patients with DM undergoing CABG

**Figure 2:**
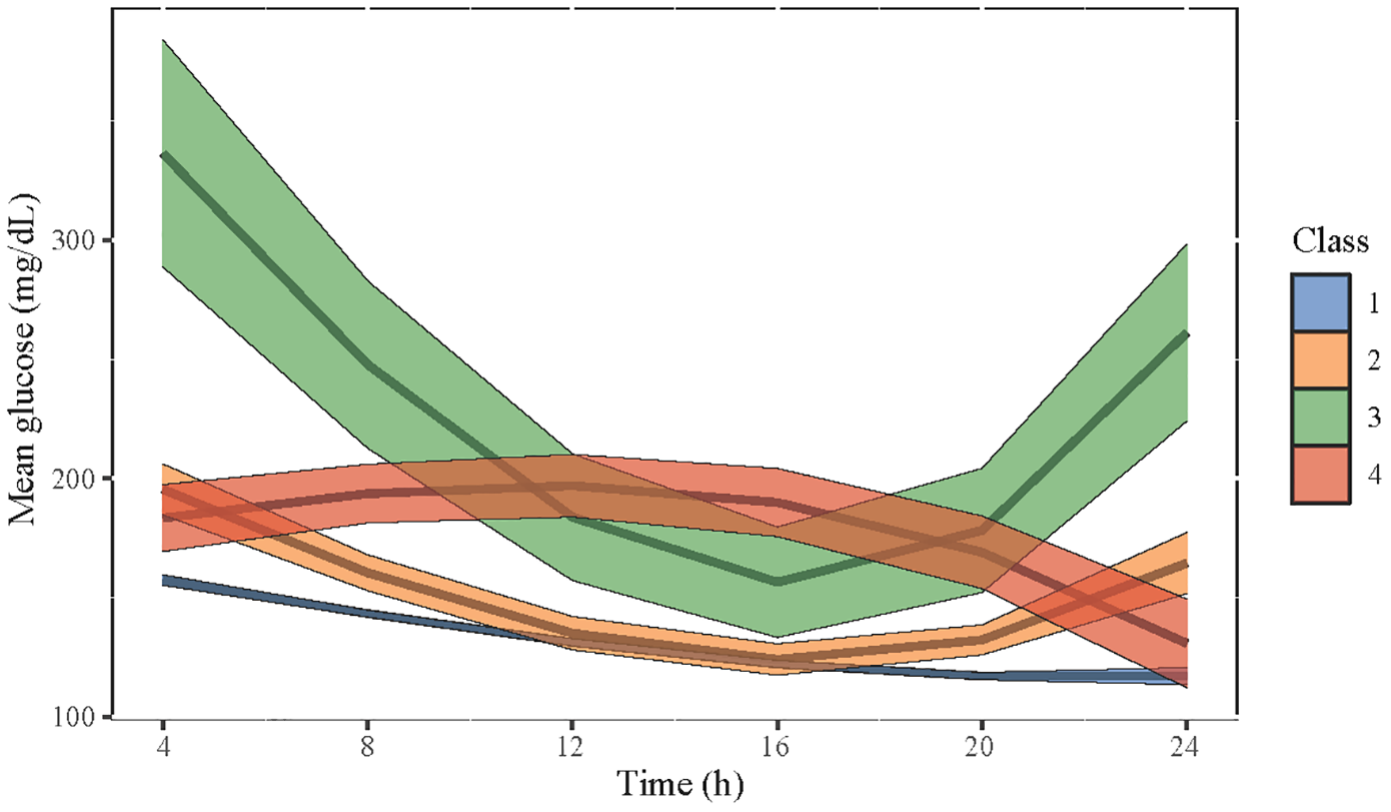
The four selected classes of trajectory patterns of MBG in patients with DM who underwent CABG. Class 1 is in blue, class 2 is in yellow, class 3 is in green, and class 4 is in red. The width of each color band represents the confidence interval of the predictive value of MBG.

We further selected the trajectory classes of blood glucose in LGMM. **Supplementary Tables S3**, **S4** respectively show the determination of the number of classes and the average posterior probability of selected classes. We initially set the number of classes into 1–5, and according to the log likelihood, AIC, BIC, and the proportion of different classes for each condition, the LGMM had the best fitness when divided into four classes. **Figure 2** shows four different classes of trajectory patterns of MBG in patients with DM undergoing CABG. Obviously, the class 1 trajectory of MBG had the lowest baseline blood glucose and a steady downward trend within 24 h. The class 2 and class 3 trajectories of MBG had a similar tendency that firstly declined and then rose; however, the baseline and final blood glucose levels in class 3 were much higher than those in class 2. In contrast, although class 4 MBG trajectory had a similar baseline blood glucose level to that in class 2, it had a tendency to slightly increase and then decrease.

**Figure 3:**
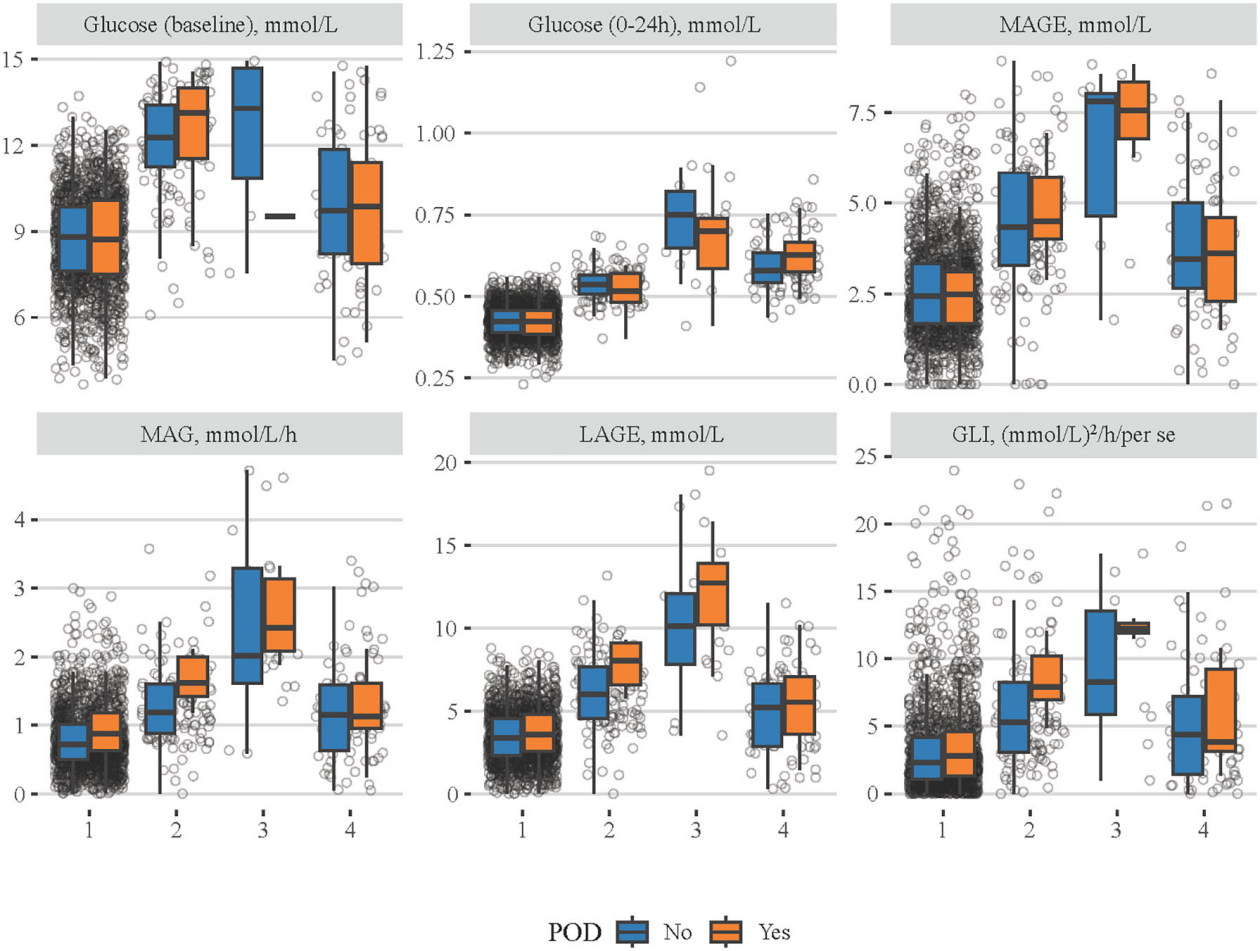
Comparisons of different blood glucose-related indexes among patients with four classes of MBG trajectories. The blue boxes represent the non-POD group and the orange boxes denote the POD group.

The comparisons of different blood glucose indexes among patients with the four classes of MBG trajectory are shown in **Figure 3**, and details on statistical values are shown in **Supplementary Table S5**. It seemed that patients in class 1 had the lowest levels of baseline blood glucose, MBG, MAGE, MAG, LAGE, and GLI, while those in class 3 had the highest ones. In addition, the associations of four classes of MBG trajectories with POD were investigated (**Table 4**). After adjusting for all covariates, patients with class 3 (OR = 3.197, 95% CI: 1.095–9.339) or class 4 (OR = 2.987, 95% CI: 1.618–5.514) MBG trajectory seemed to have higher odds of POD, compared to those with class 1 MBG trajectory.

**Table 4:** Associations of four classes of MBG trajectories with POD.

Classes	Model 1		Model 2		Model 3		Model 4		Model 5	
	OR (95% CI)	*p*	OR (95% CI)	*p*	OR (95% CI)	*p*	OR (95% CI)	*p*	OR (95% CI)	*p*
1	Ref		Ref		Ref		Ref		Ref	
2	0.960 (0.492–1.873)	0.904	0.852 (0.425–1.707)	0.651	0.822 (0.404–1.675)	0.590	0.877 (0.433–1.775)	0.714	0.867 (0.426–1.765)	0.694
3	4.102 (1.696–9.925)	0.002	2.857 (1.062–7.684)	0.038	3.053 (1.086–8.581)	0.034	3.348 (1.169–9.588)	0.024	3.197 (1.095–9.339)	**0.034**
4	3.712 (2.224–6.195)	<0.001	2.853 (1.624–5.010)	<0.001	2.868 (1.563–5.262)	<0.001	3.089 (1.680–5.679)	<0.001	2.987 (1.618–5.514)	**<0.001**

MBG, mean blood glucose; POD, postoperative delirium; OR, odds ratio; CI, confidence interval; Ref, reference.

Model 1: unadjusted model.

Model 2: adjusted for age, HR, and SpO_2_.

Model 3: adjusted for age, HR, SpO_2_, RDW, hematocrit, eGFR, INR, PT, BUN, bicarbonate, and Na.

Model 4: adjusted for age, HR, SpO_2_, RDW, hematocrit, eGFR, INR, PT, BUN, bicarbonate, Na, SOFA, and CCI.

Model 5: adjusted for age, HR, SpO_2_, RDW, hematocrit, eGFR, INR, PT, BUN, bicarbonate, Na, SOFA, CCI, sepsis, CKD, mechanical ventilation use, and vasopressor use.

The bold values provided means statistically significant (namely P<0.05).

### Potential interaction effects between blood glucose and hepatorenal function on POD

Moreover, we assessed the potential interaction effects between blood glucose (MBG, MAG, and GLI) and hepatorenal function (INR and eGFR) on POD (**Table 5**), to explore the possible metabolic pathway in which blood glucose influences POD risk in patients with DM who received CABG. The results showed that MAG and INR had a potential synergistic effect on POD, with a RERI of 0.71 (95% CI: 0.14–1.27) and an AP of 0.71 (95% CI: 0.12–1.19). GLI also had a potential synergistic effect with INR on POD, with a RERI of 0.78 (95% CI: 0.19–1.39) and an AP of 0.69 (95% CI: 0.16–1.12).

**Table 5:** Potential interaction effects between glucose fluctuation indexes and hepatorenal function on POD.

Interaction effects	OR (95% CI)	*p*	Interaction statistics	Estimate (95% CI)
MBG*INR				
Low MBG & Low INR	Ref		RERI	0.09 (-0.82, 0.76)
High MBG & Low INR	1.438 (0.900–2.297)	0.128		
Low MBG & High INR	1.663 (0.992–2.789)	0.054	AP	0.08 (-0.75, 0.59)
High MBG & High INR	1.972 (1.183–3.290)	**0.009**		
MAG*INR				
Low MAG & Low INR	Ref		RERI	**0.71 (0.14, 1.27)**
High MAG & Low INR	3.281 (1.936–5.562)	**<0.001**		
Low MAG & High INR	3.287 (1.788–6.042)	**<0.001**	AP	**0.71 (0.12, 1.19)**
High GLI & High INR	3.234 (1.805–5.794)	**<0.001**		
GLI*INR				
Low GLI & Low INR	Ref		RERI	**0.78 (0.19, 1.39)**
High GLI & Low INR	1.438 (0.900–2.297)	0.128		
Low GLI & High INR	1.663 (0.992–2.789)	0.054	AP	**0.69 (0.16, 1.12)**
High GLI & High INR	1.972 (1.183–3.290)	**0.009**		
MBG*eGFR				
Low MBG & High eGFR	Ref		RERI	0.24 (−0.67, 0.95)
High MBG & High eGFR	1.368 (0.903–2.075)	0.140		
Low MBG & Low eGFR	1.441 (0.781–2.661)	0.242	AP	0.22 (−0.71, 0.8)
High MBG & Low eGFR	1.487 (0.775–2.853)	0.233		
MAG*eGFR				
Low MAG & High eGFR	Ref		RERI	−0.42 (−1.46, 0.23)
High MAG & High eGFR	1.578 (1.040–2.393)	**0.032**		
Low MAG & Low eGFR	0.960 (0.461–1.996)	0.912	AP	−0.68 (−2.98, 0.36)
High MAG & Low eGFR	2.193 (1.191–4.040)	**0.012**		
GLI*eGFR				
Low GLI & High eGFR	Ref		RERI	−0.39 (−1.46, 0.33)
High GLI & High eGFR	1.368 (0.903–2.075)	0.140		
Low GLI & Low eGFR	1.441 (0.781–2.661)	0.242	AP	−0.53 (−2.48, 0.33)
High GLI & Low eGFR	1.487 (0.775–2.853)	0.233		

POD, postoperative delirium; OR, odds ratio; CI, confidence interval; MBG, mean blood glucose; INR, international normalized ratio; Ref, reference; RERI, relative excess risk due to interaction; AP, attributable proportion of interaction; MAG, mean absolute glucose; GLI, glycemic lability index.

The bold values provided means statistically significant (namely P<0.05).

## Discussion

The present Mendelian randomization (MR) study investigated the potential causal associations of blood glucose-related indexes and MBG trajectories with POD in patients with DM undergoing CABG. The results showed that higher levels of MBG, MAG, and GLI were linked to higher odds of POD. Similarly, these positive associations were observed in patients aged <65 years old, male patients, White patients, those with eGFR <60 and INR <1.5, patients with sepsis, and those who received mechanical ventilation and vasopressors. Patients with class 3 and class 4 MBG trajectory seemed to have higher odds of POD compared to those with class 1 MBG trajectory. In addition, there were potential synergistic effects between MAG and INR and between GLI and INR, on POD.

To our knowledge, it was the first time to discuss the associations of different blood glucose indexes and MBG trajectories with POD in patients with DM undergoing CABG. Patients with DM are prone to a diffuse and rapidly progressive form of atherosclerosis, and the superiority of CABG—greater survival, fewer recurrent infarctions, and less need for re-intervention—has been demonstrated (23). However, it is also important for these patients’ management of blood glucose, which may be linked to the risk of POD. According to our findings, although the blood glucose level upon ICU admission was not significantly associated with POD, higher levels of MBG and MAG in 24 h was linked to higher odds of POD. Moreover, compared to LAGE, GLI within 24 h after ICU admission seemed to be more significant for those assessed with POD. Gandhi et al. (24) suggested that intraoperative hyperglycemia (the initial, mean, and maximal intraoperative glucose concentrations) is an independent risk factor for complications, including delirium, after cardiac surgery. Another retrospective cohort study conducted by Choi et al. (13) also considered that patients with a higher intraoperative GV have an increased risk of POD. Compared with previous studies, the current research included patients from the MIMIC-IV database and provided information on the relationship between postoperative blood glucose and POD. In fact, conclusions on the role of blood glucose in POD are still inconsistent. A systematic review and meta-analysis showed no significant difference in POD between conventional glucose control and intensive glucose control in patients with diabetes (25). van Keulen et al. (9) found a positive association between delirium and hypoglycemia in critically ill patients with DM, but delirium was not linked to more pronounced glucose variability. Although this study similarly showed no significant association between LAGE and POD to van Keulen’s, the MBG and MAG had positive associations with POD. Therefore, further studies are still needed to clarify the causal association between blood glucose change and POD in patients with DM undergoing CABG.

The exact mechanisms by which blood glucose influences POD in patients with diabetes undergoing CABG are not well understood and have been proposed to play a key role in the development of postoperative neurocognitive disorders including POD (26). The brain is particularly sensitive to blood glucose concentrations. The brain accounts for 50% of the body’s glucose consumption and lacks the ability to utilize other forms of energy, and the metabolism and homeostasis of blood glucose levels were controlled by glucose-sensing neurons presented in the brain (27). Hyperglycemia as both a result and a cause of perioperative inflammation (28, 29) can cause oxidative stress and result in neuronal damage and cognitive impairment (30). Therefore, because neuroinflammation is an important mechanism for the development of POD and, in turn, neuroinflammation can be caused by systemic inflammation that is closely interlinked to glucose homeostasis, the relationship between hyperglycemia and POD is evident (31, 32). Herein, these findings suggested that blood glucose levels (both MBG and GLI) should be monitored more closely in patients with diabetes who underwent CABG to prevent delirium. Furthermore, liver and kidney as important metabolic organs become functionally impaired due to any metabolic disorder in patients with DM (33). Studies showed that disorder of the gut–liver–brain axis influences disease development and progression, including choline, blood–brain barrier, vagus nerve, and neurotransmitters (34). Ghoshal et al. (35) concluded that the presence of higher albuminuria and lower eGFR contributes to the relationship between nephropathy and cognitive impairment in patients with diabetic kidney disease. Similarly in the current study, there were potential synergistic effects between MAG and INR and between GLI and INR, on POD, which may further suggest that blood glucose change in patients with DM was associated with POD risk with the potential mechanism of metabolic disorders associated with changes in liver and kidney function.

We also assessed the odds of POD in patients with different MBG trajectories. Among the four selected classes, it seemed that patients with class 3 or class 4 MBG trajectory had higher odds of POD, compared to those with class 1 MBG trajectory. No study has reported the role of MBG trajectory in POD among patients who underwent CABG. According to our results, although patients with class 3 and class 4 MBG trajectory had contrasting tendencies, they were both potential risk factors for POD. Specifically, class 3 had a very high baseline MBG, and clinicians may recommend treatment to reduce the MBG level rapidly, which recovered after a while. Tight glucose control has been linked to increased hypoglycemia rates and increased delirium rates, which may be due to the fact that insulin-induced hypoglycemia affects brain function (12, 36). Hence, if patients with DM who underwent CABG had high levels of blood glucose upon ICU admission, rapid glucose reduction could not be a good option in clinical practice. Moreover, class 4 represented a relatively low level of blood glucose, but no hypoglycemic measures were taken in the following period of time, and although the MBG level was subsequently reduced, it was also associated with higher odds of POD. It indicated that although patients have no severe hyperglycemia, hypoglycemic measures should be implemented to decrease the MBG level within 24 h after ICU admission to reduce the risk of POD. Herein, in clinical practice, stably reducing blood glucose levels within 24 h after ICU admission and keeping fluctuations in blood glucose levels as low as possible could be associated with reducing subsequent POD risk.

In addition, the positive associations of MBG, MAG, and GLI with POD were observed in patients aged <65 years old, male patients, White patients, those with eGFR <60 and INR <1.5, patients with sepsis, and those who received mechanical ventilation and vasopressors. DM and older age have been reported to be preoperative risk factors of developing delirium post‐CABG (37). Older age is a predictor for POD in the general population after cardiac surgery (38). Old patients with lower cognitive function and organ function reserve have a decreased ability to maintain normal brain function when under perioperative stress generally. Our results indicated that controlling blood glucose is also important in patients younger than 65 years old. Wittmann et al.’s (39) results showed that women after a cardiac procedure showed a 52% POD risk, while men had a 64% POD risk. Although it seemed that measurement of MBG, MAG, and GLI may be more significant in male patients with DM than in female patients according to our results, gender differences in POD are still poorly considered. INR has been reported to be one of the risk factors of POD after liver transplantation (40). Herein, the associations of blood glucose-related indexes with POD were significant only in patients with INR <1.5, which may be explained by the dynamic detection of liver function, and appropriate measures were taken by clinicians towards patients who had an abnormal INR value after the procedure. It has been widely recognized that delirium can be a symptom of end organ dysfunction in sepsis. Differently, Martin et al. (41) considered an association between delirium and post-operative sepsis in the CABG population, and delirium emerged as an independent predictor of sepsis. According to our findings, it seemed that blood glucose may be involved in the association between sepsis and delirium; however, the causal relationship between them needs further clarification. In addition, among patients who received mechanical ventilation or who were treated with vasopressors, focusing on the levels of MAG, MBG, and GLI may be important for the reduction of POD risk. A stable blood glucose level during the preoperative and late postoperative periods could predispose patients to decreased infectious complications and lower morbidity and mortality rates (42, 43).

The present study is based on the large public clinical database MIMIC-IV, which includes a large sample of real cases and is relatively representative of the United States population. By discussing the association of blood glucose-related indexes and blood glucose trajectories with POD in patients with DM who underwent CABG, this study may provide some references for management of blood glucose and prevention of delirium in this population. This study performed a comprehensive collection of blood glucose-related indicators, from the average level, instability, trajectory changes, and other aspects of analysis. Furthermore, information on multi-dimensional influencing factors, such as demographic data, vital signs, laboratory index, and complications, was gathered. However, there are still some limitations. Owing to the retrospective nature of the study, selection bias was inevitable. Moreover, patients in the MIMIC-IV database were from a single center, and therefore, multicenter prospective cohorts are needed to explore the causal relationship of blood glucose with POD in patients with DM who underwent CABG.

## Conclusion

Clinicians need to focus on blood glucose-related indexes, including MBG, MAG, GLI, and trajectory change, which were all associated with POD, to reduce the risk of delirium in patients with DM who underwent CABG. Stably reducing blood glucose levels within 24 h after ICU admission and keeping fluctuations in blood glucose levels as low as possible could be associated with reducing subsequent POD risk.

## Data availability statement

The datasets presented in this study can be found in online repositories. The names of the repository/repositories and accession number(s) can be found in the article/**Supplementary Material**.

## Ethics statement

The studies involving humans were approved by the Institutional Review Board of Beijing Chao-Yang Hospital. The studies were conducted in accordance with the local legislation and institutional requirements. Written informed consent for participation was not required from the participants or the participants’ legal guardians/next of kin in accordance with the national legislation and institutional requirements.

## Author contributions

FW: Writing – original draft. XM: Writing – review & editing.
