# Supplementary material for: Association of blood glucose change with postoperative delirium after coronary artery bypass grafting in patients with diabetes mellitus: a study of the MIMIC-IV database
Source: Front Endocrinol (Lausanne). 2024 Jun 20;15:1400207. doi: 10.3389/fendo.2024.1400207 (PMC11222311; doi:10.3389/fendo.2024.1400207)
Supplement: Supplementary file 2 [file DataSheet_2.docx]

**Table S1. Comparation between non-POD group and POD group on different blood glucose indexes.**

| POD | Variables | Minimum | Maximum | Median | Q_1_ | Q_3_ |
| --- | --- | --- | --- | --- | --- | --- |
| Non | Glucose (baseline), mmol/L | 3.667 | 14.944 | 8.963 | 7.685 | 10.111 |
| Non | Glucose (0-24h), mmol/L | 0.231 | 1.221 | 0.432 | 0.393 | 0.475 |
| Non | MAGE, mmol/L | 0 | 8.926 | 2.569 | 1.732 | 3.556 |
| Non | MAG, mmol/L/h | 0.006 | 4.713 | 0.757 | 0.522 | 1.101 |
| Non | LAGE, mmol/L | 0.037 | 19.5 | 3.583 | 2.428 | 4.833 |
| Non | GLI, (mmol/L)^2^/h/per se | 0 | 23.943 | 2.509 | 1.178 | 4.753 |
| Yes | Glucose (baseline), mmol/L | 3.875 | 14.778 | 9.014 | 7.5 | 10.403 |
| Yes | Glucose (0-24h), mmol/L | 0.293 | 0.901 | 0.442 | 0.397 | 0.497 |
| Yes | MAGE, mmol/L | 0 | 8.833 | 2.628 | 1.866 | 3.623 |
| Yes | MAG, mmol/L/h | 0.017 | 3.574 | 0.966 | 0.703 | 1.355 |
| Yes | LAGE, mmol/L | 0.1 | 16.389 | 4 | 2.708 | 5.546 |
| Yes | GLI, (mmol/L)^2^/h/per se | 0.001 | 21.495 | 3.22 | 1.694 | 5.354 |

POD: postoperative delirium, Q₁: 1st quartile, Q₃: 3rd quartile, MAG: mean absolute glucose, MAGE: mean amplitude of glycemic excursions, GLI: glycemic lability index.

**Table S2. Covariates associated with POD in DM patients underwent CABG.**

| Variables | OR (95% CI) | *P* |
| --- | --- | --- |
| Age | 1.511 (1.280-1.782) | **<0.001** |
| Gender |  |  |
| Female | Ref |  |
| Male | 0.737 (0.529-1.027) | 0.072 |
| Race |  |  |
| Other | Ref |  |
| White | 0.894 (0.645-1.238) | 0.499 |
| Insurance |  |  |
| Medicaid | Ref |  |
| Medicare | 1.149 (0.538-2.456) | 0.719 |
| Other | 0.730 (0.339-1.574) | 0.423 |
| HR | 1.485 (1.286-1.714) | **<0.001** |
| DBP | 0.896 (0.765-1.050) | 0.176 |
| SBP | 0.947 (0.811-1.107) | 0.496 |
| Temperature | 0.963 (0.828-1.121) | 0.629 |
| SpO_2_ | 0.755 (0.674-0.846) | **<0.001** |
| pH | 0.869 (0.747-1.011) | 0.069 |
| WBC | 1.058 (0.932-1.200) | 0.384 |
| RDW | 1.388 (1.222-1.577) | **<0.001** |
| Platelet | 0.922 (0.786-1.082) | 0.321 |
| Hematocrit | 0.702 (0.595-0.828) | **<0.001** |
| eGFR | 0.606 (0.526-0.699) | **<0.001** |
| INR | 1.283 (1.082-1.522) | **0.004** |
| PT | 1.220 (1.016-1.464) | **0.033** |
| BUN | 1.367 (1.213-1.541) | **<0.001** |
| Bicarbonate | 0.639 (0.548-0.745) | **<0.001** |
| Na | 0.820 (0.702-0.958) | **0.012** |
| K | 1.142 (0.983-1.325) | 0.082 |
| Chloride | 0.919 (0.693-1.219) | 0.559 |
| SOFA | 1.593 (1.387-1.829) | **<0.001** |
| CCI | 1.893 (1.644-2.179) | **<0.001** |
| GCS | 1.037 (0.884-1.217) | 0.652 |
| Sepsis |  |  |
| No | Ref |  |
| Yes | 1.529 (1.124-2.079) | **0.007** |
| CVD |  |  |
| No | Ref |  |
| Yes | 1.577 (0.952-2.610) | 0.077 |
| CKD |  |  |
| No | Ref |  |
| Yes | 2.103 (1.514-2.922) | **<0.001** |
| Liver disease |  |  |
| No | Ref |  |
| Yes | 1.475 (0.720-3.023) | 0.288 |
| Depression |  |  |
| No | Ref |  |
| Yes | 0.602 (0.260-1.393) | 0.235 |
| Mechanical ventilation status |  |  |
| Yes | Ref |  |
| No | 0.382 (0.264-0.553) | **<0.001** |
| Vasopressors use |  |  |
| No | Ref |  |
| Yes | 4.277 (2.237-8.179) | **<0.001** |
| Sedative drug use |  |  |
| No | Ref |  |
| Yes | 1.714 (0.529-5.552) | 0.369 |
| Antibiotic drug use |  |  |
| No | Ref |  |
| Yes | 5.953 (0.820-43.234) | 0.078 |

POD: postoperative delirium, DM: diabetes mellitus, CABG: coronary artery bypass grafting, OR: odds ratio, CI: confidence interval, Ref: reference, HR: heart rate, DBP: diastolic blood pressure, SBP: systolic blood pressure, WBC: white blood cell, RDW: red cell distribution width, eGFR: estimated glomerular filtration rate, INR: international normalized ratio, PT: prothrombin time, BUN: blood urea nitrogen, Na: sodium, K: potassium, SOFA: Sequential Organ Failure Assessment, CCI: Charlson Comorbidity Index, GCS: Glasgow Coma Scale, CVD: cardiovascular disease, CKD: chronic kidney disease.

**Table S3. Determination of the number of classes in LGMM.**

| Number of classes | Log likelihood | AIC | BIC | Entropy | Class 1 proportion | Class 2 proportion | Class 3 proportion | Class 4 proportion | Class 5 proportion |
| --- | --- | --- | --- | --- | --- | --- | --- | --- | --- |
| 1 | -38436.92 | 76887.84 | 76927.21 | 1 | 100% |  |  |  |  |
| 2 | -37941.91 | 75907.81 | 75975.29 | 0.81 | 93.35% | 6.65% |  |  |  |
| 3 | -37814.07 | 75662.14 | 75757.73 | 0.83 | 91.74% | 2.40% | 5.87% |  |  |
| **4** | **-37775.45** | **75594.90** | **75718.60** | **0.72** | **86.80%** | **6.55%** | **1.56%** | **5.09%** |  |
| 5 | -37753.76 | 75561.52 | 75713.35 | 0.68 | 81.08% | 7.00% | 1.47% | 2.89% | 7.588% |

LGMM: latent growth mixture modeling, AIC: Akaike information criterion, BIC: Bayesian information criterion.

**Table S4. Average posterior probability of the selected classes in LGMM.**

| Classes | Probability 1 | Probability 2 | Probability 3 | Probability 4 |
| --- | --- | --- | --- | --- |
| 1 | **0.8800** | 0.0939 | 0.0015 | 0.0246 |
| 2 | 0.1928 | **0.7071** | 0.0289 | 0.0712 |
| 3 | 0.0069 | 0.0464 | **0.9047** | 0.0420 |
| 4 | 0.0816 | 0.0871 | 0.0359 | **0.7954** |

LGMM: latent growth mixture modeling.

**Table S5. Comparation among four classes of MBG trajectory on different blood glucose indexes.**

| POD | Variables | Class | Minimum | Maximum | Median | Q_1_ | Q_3_ |
| --- | --- | --- | --- | --- | --- | --- | --- |
| No | Glucose (baseline), mmol/L | 1 | 3.667 | 13.722 | 8.815 | 7.623 | 9.852 |
| No | Glucose (0-24h), mmol/L | 1 | 0.231 | 0.59 | 0.423 | 0.389 | 0.457 |
| No | MAGE, mmol/L | 1 | 0 | 8 | 2.444 | 1.677 | 3.34 |
| No | MAG, mmol/L/h | 1 | 0.009 | 2.997 | 0.722 | 0.503 | 1.012 |
| No | LAGE, mmol/L | 1 | 0.056 | 8.5 | 3.389 | 2.333 | 4.542 |
| No | GLI, (mmol/L)^2^/h/per se | 1 | 0 | 23.943 | 2.309 | 1.089 | 4.197 |
| Yes | Glucose (baseline), mmol/L | 1 | 3.875 | 12.522 | 8.722 | 7.5 | 10.069 |
| Yes | Glucose (0-24h), mmol/L | 1 | 0.293 | 0.559 | 0.423 | 0.385 | 0.457 |
| Yes | MAGE, mmol/L | 1 | 0 | 7.407 | 2.472 | 1.667 | 3.101 |
| Yes | MAG, mmol/L/h | 1 | 0.017 | 2.713 | 0.88 | 0.639 | 1.185 |
| Yes | LAGE, mmol/L | 1 | 0.1 | 8.806 | 3.583 | 2.589 | 4.815 |
| Yes | GLI, (mmol/L)^2^/h/per se | 1 | 0.001 | 20.275 | 2.796 | 1.325 | 4.62 |
| No | Glucose (baseline), mmol/L | 2 | 6.083 | 14.917 | 12.278 | 11.264 | 13.407 |
| No | Glucose (0-24h), mmol/L | 2 | 0.442 | 0.685 | 0.537 | 0.507 | 0.565 |
| No | MAGE, mmol/L | 2 | 0 | 8.926 | 4.333 | 3.282 | 5.838 |
| No | MAG, mmol/L/h | 2 | 0.006 | 3.179 | 1.188 | 0.881 | 1.605 |
| No | LAGE, mmol/L | 2 | 0.037 | 11.685 | 6.023 | 4.583 | 7.691 |
| No | GLI, (mmol/L)^2^/h/per se | 2 | 0 | 22.926 | 5.281 | 3.1 | 8.219 |
| Yes | Glucose (baseline), mmol/L | 2 | 7 | 14.556 | 13.139 | 11.55 | 14.01 |
| Yes | Glucose (0-24h), mmol/L | 2 | 0.371 | 0.594 | 0.517 | 0.482 | 0.571 |
| Yes | MAGE, mmol/L | 2 | 2.881 | 6.917 | 4.505 | 4.005 | 5.715 |
| Yes | MAG, mmol/L/h | 2 | 1.185 | 3.574 | 1.625 | 1.43 | 1.999 |
| Yes | LAGE, mmol/L | 2 | 5.761 | 13.167 | 8.067 | 6.611 | 9.098 |
| Yes | GLI, (mmol/L)^2^/h/per se | 2 | 4.854 | 12.063 | 7.933 | 6.994 | 10.192 |
| No | Glucose (baseline), mmol/L | 3 | 7.542 | 14.944 | 13.292 | 10.865 | 14.694 |
| No | Glucose (0-24h), mmol/L | 3 | 0.538 | 1.221 | 0.749 | 0.649 | 0.822 |
| No | MAGE, mmol/L | 3 | 1.778 | 8.556 | 7.806 | 4.646 | 8.035 |
| No | MAG, mmol/L/h | 3 | 0.593 | 4.713 | 2.014 | 1.615 | 3.285 |
| No | LAGE, mmol/L | 3 | 3.556 | 19.5 | 10.111 | 7.833 | 12.083 |
| No | GLI, (mmol/L)^2^/h/per se | 3 | 0.988 | 17.794 | 8.262 | 5.899 | 13.539 |
| Yes | Glucose (baseline), mmol/L | 3 | 9.537 | 9.537 | 9.537 | 9.537 | 9.537 |
| Yes | Glucose (0-24h), mmol/L | 3 | 0.41 | 0.901 | 0.699 | 0.585 | 0.739 |
| Yes | MAGE, mmol/L | 3 | 6.278 | 8.833 | 7.565 | 6.771 | 8.354 |
| Yes | MAG, mmol/L/h | 3 | 1.88 | 3.324 | 2.42 | 2.086 | 3.137 |
| Yes | LAGE, mmol/L | 3 | 7.111 | 16.389 | 12.722 | 10.222 | 13.907 |
| Yes | GLI, (mmol/L)^2^/h/per se | 3 | 11.502 | 12.969 | 12.236 | 11.869 | 12.602 |
| No | Glucose (baseline), mmol/L | 4 | 4.5 | 14.556 | 9.718 | 8.222 | 11.847 |
| No | Glucose (0-24h), mmol/L | 4 | 0.436 | 0.858 | 0.579 | 0.542 | 0.633 |
| No | MAGE, mmol/L | 4 | 0 | 8.574 | 3.456 | 2.649 | 5.014 |
| No | MAG, mmol/L/h | 4 | 0.056 | 3.398 | 1.155 | 0.632 | 1.597 |
| No | LAGE, mmol/L | 4 | 0.333 | 11.5 | 5.243 | 2.903 | 6.667 |
| No | GLI, (mmol/L)^2^/h/per se | 4 | 0.008 | 18.313 | 4.406 | 1.421 | 7.225 |
| Yes | Glucose (baseline), mmol/L | 4 | 5.148 | 14.778 | 9.861 | 7.889 | 11.403 |
| Yes | Glucose (0-24h), mmol/L | 4 | 0.493 | 0.771 | 0.626 | 0.574 | 0.667 |
| Yes | MAGE, mmol/L | 4 | 1.5 | 7.833 | 3.611 | 2.296 | 4.611 |
| Yes | MAG, mmol/L/h | 4 | 0.25 | 3.066 | 1.133 | 0.954 | 1.614 |
| Yes | LAGE, mmol/L | 4 | 1.5 | 10.167 | 5.556 | 3.62 | 7.083 |
| Yes | GLI, (mmol/L)^2^/h/per se | 4 | 1.355 | 21.495 | 3.814 | 3.124 | 9.24 |

MBG: mean blood glucose, POD: postoperative delirium, Q₁: 1st quartile, Q₃: 3rd quartile, MAG: mean absolute glucose, MAGE: mean amplitude of glycemic excursions, GLI: glycemic lability index.
